# Co‑occurring mental and substance use disorders among residents of Drug Addiction Rehabilitation Centers (DARCs) in Japan: Characterizing dual‑diagnosis profiles

**DOI:** 10.1002/pcn5.70196

**Published:** 2025-09-24

**Authors:** Satomi Mizuno, Takuya Shimane, Satoshi Inoura, Toshihiko Matsumoto

**Affiliations:** ^1^ Department of Drug Dependence Research, National Institute of Mental Health National Center of Neurology and Psychiatry Kodaira Tokyo Japan; ^2^ Department of Nursing Faculty of Nursing Niigata Seiryo University Niigata Japan

**Keywords:** Drug Addiction Rehabilitation Centers, drug rehabilitation support facilities, dual diagnosis, mental disorder, substance use disorder

## Abstract

**Aim:**

The co‐occurrence of substance use and mental disorders, commonly referred to as a dual diagnosis, presents considerable challenges to the recovery process. Despite its clinical relevance, only a few studies have examined the characteristics correlated with dual diagnoses among individuals utilizing rehabilitation services. Thus, in this study, we aimed to identify the factors associated with dual diagnoses in study participants receiving care at drug rehabilitation support facilities.

**Methods:**

We analyzed data from participants receiving care at drug rehabilitation support facilities, specifically the Drug Addiction Rehabilitation Centers. Multivariable logistic regression analysis was performed to examine the correlations among dual diagnoses and sociodemographic characteristics, facility utilization patterns, substance dependence, physical health, and recovery outcomes at the 1‐year follow‐up.

**Results:**

Dual diagnosis was notably correlated with the female sex (adjusted odds ratio [AOR] = 4.18, 95% confidence interval [CI]: 2.01–8.67), history of incarceration (AOR = 2.10, 95% CI: 1.35–3.28), and prior treatment for substance use disorder (AOR = 2.22, 95% CI: 1.30–3.91). At the 1‐year follow‐up, participants with dual diagnoses exhibited poor recovery outcomes across multiple domains; they were more likely to be unemployed (AOR = 2.10, 95% CI: 1.35–3.31) and had greater difficulty maintaining drug abstinence (AOR = 1.85, 95% CI: 1.17–2.94).

**Conclusion:**

Individuals with dual diagnoses were more likely to be female, have histories of incarceration, and have prior treatment experiences. After 1 year, they had poorer outcomes in terms of employment and drug abstinence, highlighting the need for tailored support in recovery programs.

## INTRODUCTION

A dual diagnosis, defined as the co‐occurrence of a substance use disorder and at least one mental disorder, represents a key challenge in addiction treatment. Patients with dual diagnoses present with concurrent conditions, such as depression, anxiety, schizophrenia, or developmental disorders, which complicate treatment and increase the risk of relapse.^1^, ^2^, ^3^, ^4^, ^5^, ^6^, ^7^ Severe psychiatric symptoms, economic hardship, and social isolation can hinder access to continued care.^8^, ^9^, ^10^, ^11^, ^12^, ^13^, ^14^ In response, increasing attention has been directed toward integrated models of support that combine mental healthcare services, addiction treatment, and social services, thereby addressing the limitations of traditionally fragmented systems.^15^, ^16^, ^17^, ^18^ Effective management of a dual diagnosis requires a comprehensive approach that considers both medical and psychosocial dimensions.

Large‐scale epidemiological studies have identified patterns of dual diagnoses within the general population.^2^, ^19^, ^20^, ^21^, ^22^, ^23^ This condition is more common in male patients, who frequently present with co‐occurring conditions such as antisocial personality disorder or schizophrenia, whereas female patients more commonly experience depression or anxiety.^2^ Mental disorders commonly precede substance use; factors such as an early onset, trauma, economic hardship, and social isolation have been associated with an increased risk.^15^, ^19^, ^20^, ^21^ Prevalence varies by race, with higher rates reported among Caucasian populations and lower rates among Asian populations.^23^ Female patients with dual diagnoses generally have histories of physical abuse and face challenges related to parenting.^3^, ^22^ Moreover, these conditions are associated with complex health and social difficulties.^6^

Epidemiological studies have contributed to a shift in understanding a dual diagnosis as a broader social concern rather than an individual issue, thereby influencing the development of integrated support systems.^2^, ^19^, ^20^, ^21^, ^22^, ^23^ Nevertheless, these studies have typically focused on noninstitutionalized individuals at the time of data collection, excluding those receiving care in psychiatric hospitals, residing in correctional facilities, or living in recovery support centers. Consequently, the experiences of patients with dual diagnoses who face long‐term challenges and social exclusion remain underrepresented. Existing research involving institutionalized populations remains limited and has primarily concentrated on those in correctional settings.^17^, ^24^, ^25^, ^26^, ^27^, ^28^

There is a lack of research regarding patients residing in intermediate support settings, such as drug rehabilitation facilities, with a focus on the specific challenges experienced by those with dual diagnoses. Additionally, international studies^15^, ^17^, ^19^, ^20^, ^21^, ^22^, ^23^, ^24^, ^25^, ^26^, ^27^, ^28^ have demonstrated that factors including sex, age, severity of mental illness, involvement within the justice system, and institutional frameworks can influence both the development of dual diagnoses and recovery trajectory. However, research in Japan incorporating these perspectives remains scarce, and evidence grounded in the field of support is limited.^29^

In Japan, drug recovery support facilities, such as the Drug Addiction Rehabilitation Centers (DARCs), play an essential role in supporting the reintegration of individuals with substance use disorders. These facilities generally work in coordination with the system, involving partial suspension of sentence execution.^29^, ^30^, ^31^, ^32^, ^33^, ^34^, ^35^ DARCs are community‐based rehabilitation facilities based on the well‐known 12‐step program and operated by individuals in recovery. The 12‐step program is a structured, peer‐support‐based approach to help individuals recover from addiction. Participants acknowledge personal limitations, rely on a “higher power,” reflect on past behavior, and rebuild their lives through mutual support.^34^, ^36^ These centers function as intermediate support settings, providing residents with a stable living environment and structured pathways toward social reintegration.^30^, ^31^, ^32^, ^33^, ^34^, ^35^, ^37^, ^38^ Due to the limited availability of addiction treatment services in Japan, DARCs serve as vital support hubs. However, there is limited information regarding the experiences of individuals with dual diagnoses within these settings, and further empirical investigation is required to enhance the quality of care. Understanding the impact of dual diagnoses on individuals in underrepresented settings is crucial for developing more effective support systems. Examining these characteristics can enable the adaptation of recovery approaches and provide practical guidance for frontline practitioners.

Therefore, in this study, we aimed to identify the factors influencing dual diagnoses among individuals utilizing rehabilitation facilities in Japan. Moreover, drawing on data from individuals receiving care at DARC, we explored the correlations between dual diagnosis and sociodemographic characteristics, facility engagement, substance dependence, physical health, and recovery outcomes.

## METHODS

### Ethical considerations

The original study^29^, ^35^ was approved by the institutional ethics committee of the National Centre of Neurology and Psychiatry, Japan (approval number: A2016‐022). This study was conducted in accordance with the principles of the Declaration of Helsinki and STROBE reporting guidelines. Patients were informed regarding the purpose of the study, and those who expressed interest were invited to participate after providing written informed consent. Written informed consent was obtained from all study participants from both the original and present studies.

### Data curation

This study utilized secondary data from the “Follow‐up Study of DARC Users in Japan,” a prospective cohort study that was conducted from October 2016 to October 2021.^29^, ^35^ Details of this study have been previously described.^29^, ^35^, ^37^ In summary, participants were recruited from 46 of the 57 national DARCs between July and September 2016. Details of the study were introduced to participants by facility managers, and written informed consent was obtained before completion of the baseline questionnaire in October 2016. Follow‐up data were collected by facility staff through telephonic or in‐person interviews. Regarding this analysis, data were collected from the baseline, first follow‐up, and second follow‐up surveys, conducted at a 6‐month interval, from April to October 2017.

### Participant selection

Of the 693 eligible residents, adults fluent in Japanese who provided informed consent were included in the study. Exclusion criteria were the following: (1) intersex identification, (2) a primary addiction other than drug use, (3) missing baseline data on dual diagnosis, and (4) failure to complete the first or second follow‑up survey.

### Cohort definition

At baseline, participants were asked, “Before entering or while in the facility, have you been diagnosed with any mental disorder other than drug addiction?” Those answering “yes” were classified as the dual‑diagnosis cohort; those answering “no” or “uncertain” formed the non‑dual‑diagnosis cohort. Diagnoses included mood, psychotic, developmental, and eating disorders.

### Factors influencing dual diagnoses

In this study, we examined a range of variables, informed by previous research, to explore the factors potentially correlated with dual diagnoses.^2^, ^15^, ^17^, ^19^, ^20^, ^21^, ^22^, ^23^, ^24^, ^25^, ^26^, ^27^, ^28^ These variables included sociodemographic characteristics, facility utilization, severity of substance dependence, substance use history, physical health conditions, and 1‐year follow‐up recovery outcomes.

We first examined basic demographic characteristics from the baseline survey, including age, sex, education, employment status, and incarceration history. Furthermore, based on previous studies associating dual diagnoses with criminal involvement and financial difficulties,^26^, ^27^, ^28^ we included two additional variables: (1) legal status at the time of facility entry, such as being on probation, having a partially suspended sentence, or being under indictment, and (2) whether participants were receiving welfare.

To better understand how individuals with dual diagnoses utilize recovery facilities, we examined the following: (1) type of service provided, whether residential or day care; (2) duration of facility utilization before the baseline survey; (3) participation in rehabilitation programs; (4) interpersonal relationships within the facility; and (5) the presence of a sponsor—an individual in recovery who, as a peer rather than a professional, offers guidance, listens to concerns, and supports others in their recovery and reintegration into society.^26^, ^27^, ^28^ These variables were included due to their potential impact on social interactions and recovery outcomes, particularly for individuals facing mental health challenges.

Substance dependence severity was measured using the Drug Abuse Screening Test‐20 (DAST‐20), which classified dependence into three levels: (1) low (0–5), (2) intermediate (6–10), and (3) severe (11+).^39^ We compared the DAST‐20 scores and proportion of participants with severe dependence between the dual diagnoses and control cohorts. Additionally, we included prior opportunities for support or treatment related to substance use disorders as key variables to examine whether having dual diagnoses was correlated with differences in access to such support before facility admission.

Substance use history included the following: (1) use of illicit drugs, such as cannabis, methamphetamine, and heroin; (2) misuse of prescription drugs, such as antidepressants and antipsychotics; and (3) misuse of over‐the‐counter (OTC) medications, such as hypnotics or sedative–hypnotic medications and cough suppressants. Specifically, as individuals with dual diagnoses may self‐medicate to manage their symptoms, we aimed to capture a broad range of substance use patterns, including OTC misuse, an area that remains relatively underexplored.

We included health‐related factors, such as chronic illnesses, including diabetes mellitus and heart disease, as well as sexually transmitted and blood‐borne infections (STBBIs), such as hepatitis A, B, and C, syphilis, chlamydia, gonorrhea, and human immunodeficiency virus. These factors were considered due to individuals with substance use disorders potentially facing challenges in accessing healthcare and managing physical health.

To evaluate recovery outcomes, we assessed three indicators at the 1‐year follow‐up: (1) employment status, (2) receipt of welfare, and (3) sustained abstinence from drug use. Since dual diagnosis can make long‐term recovery more complex, we examined whether these 1‐year outcomes varied between individuals with and without dual diagnosis. The 1‐year timeframe was chosen because, although the program length varies depending on individual needs and legal circumstances, it generally spans 12–18 months, with most participants leaving the facility within 2 years to reintegrate into society. While specific practices may differ slightly across DARC locations, the first 6 months are typically considered a critical phase for rebuilding daily routines, adjusting to communal living, and reflecting on past behaviors. By 1 year, many participants have become more accustomed to the facility environment and daily structure, potentially influencing their recovery progress.

### Statistical analysis

Descriptive statistics were used to summarize baseline demographic characteristics. Differences between the dual diagnoses and control cohorts were examined using the chi‐square test or Fisher's exact test for categorical variables. For continuous variables, either the Student's *t*‐test or Mann–Whitney *U* test was applied. Continuous data were reported as means and standard deviations (SDs) and categorical data as proportions (%). To identify the characteristics correlated with dual diagnoses, a multivariable logistic regression analysis was performed. The model included variables that showed statistically significant differences in the cohort comparative analysis, in addition to clinically relevant factors, such as sex and age. Adjusted odds ratios (AORs) and 95% confidence intervals (CIs) were calculated.

Moreover, the analyses were conducted to investigate how the dual‑diagnosis status and related factors influenced key outcomes at the 1‐year follow‐up. Three separate logistic regression models were constructed using the following dependent variables: (1) employment status at 1 year (employed or unemployed), (2) receipt of public assistance or welfare at 1 year (yes or no), and (3) sustained abstinence over 1 year (yes or no). Each model included the dual‑diagnosis status as the primary independent variable. Other baseline variables that showed statistically significant correlations in the bivariate analyses were included.

Additionally, sex and age, recognized as clinically important factors, were included as covariates in all the models. AORs and 95% CIs were calculated. We aimed to evaluate whether dual diagnoses were correlated with poorer recovery outcomes and lower facility retention, while controlling for baseline characteristics, including key demographic and support‐related factors.

As the majority of recovery facility participants were male, sub‐cohort analyses by sex were conducted in addition to the primary analysis. Separate datasets were created for the male and female participants. Two‐sub‐cohort comparative and logistic regression analyses were performed to examine whether the factors were correlated with dual diagnoses varied by sex.

To assess multicollinearity in the multivariate logistic regression, variance inflation factors were calculated. Values < 10 were interpreted as indicating no multicollinearity. Statistical significance was set at *p* < 0.05.

To address multiple comparisons, we applied the Benjamini–Hochberg false discovery rate (FDR) correction across regression models. Both unadjusted and FDR‐adjusted *p*‐values are presented. Variables with FDR‐adjusted *q* < 0.05 were considered statistically significant after correction.

All analyses were performed using the R software, version 4.4.3 (R Foundation for Statistical Computing, Vienna, Austria).

## RESULTS

### Participant flow and cohort characteristics

**Figure 1:**
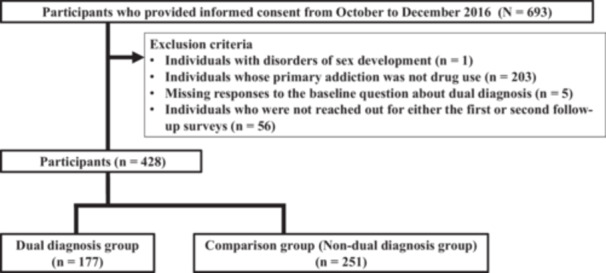
Flowchart of the study participant selection.

Among the 693 individuals initially screened, 428 adults fluent in Japanese provided informed consent and were included in the analysis (Figure 1). Reasons for exclusion were the following: intersex identification (*n* = 1), a primary addiction other than drug use (*n *= 203), missing baseline data on dual diagnoses (*n* = 9), and failure to complete either the first or second follow‑up survey (*n* = 52). Participants remained in the cohort regardless of changes in residence (e.g., discharge, reintegration, hospitalization, or facility transfer), provided they could be contacted for follow‑up.

At baseline, 177 participants who reported an additional mental disorder were assigned to the dual‑diagnosis cohort, and 251 who did not were assigned to the non‑dual‑diagnosis cohort. Reported disorders included mood (depression, bipolar), psychotic (schizophrenia), developmental (autism, attention deficit hyperactivity disorder), and eating disorders.

### Descriptive statistics regarding dual diagnoses and control cohorts

The dual‑diagnosis cohort included a significantly lower proportion of male participants than the control cohort (85.3% and 95.2%, respectively; *p* = 0.001), reflecting a higher proportion of female participants (Table 1). The dual diagnoses cohort had a higher proportion of participants with a history of incarceration (58.2% and 47.0%, respectively; *p* = 0.029) and was more likely to have received prior treatment or support for substance use disorders (87.6% and 76.9%, respectively; *p* = 0.008).

**Table 1:** Descriptive statistics of participants by dual‑diagnosis status.

	Dual‑diagnosis cohort (*n* = 177)	Non‐dual‑diagnosis (control) cohort (*n* = 251)	*p* value
Male participant	151 (85.3)	239 (95.2)	0.001
Age at entry into the facility (mean ± SD), years	38.5 ± 9.7	38.9 ± 10.4	0.712
Did not complete high school	75 (42.4)	121 (48.2)	0.274
Employment	28 (15.8)	47 (18.7)	0.516
Welfare recipient	153 (86.4)	203 (80.9)	0.166
Criminal incarceration history	103 (58.2)	118 (47.0)	0.029
Legal status at facility entry	72 (40.7)	103 (41.0)	1
Participants utilizing residential rehabilitation services	148 (83.6)	227 (90.4)	0.05
Duration of facility utilization before baseline survey (mean ± SD), months	32.7 ± 37.7	28.5 ± 35.6	0.236
Active participation in rehabilitation programs	155 (87.6)	205 (81.7)	0.131
Positive relationships with other residents and facility staff	168 (94.9)	242 (96.4)	0.606
Presence of a sponsor	140 (79.1)	195 (77.7)	0.819
Severity at facility entry: DAST‐20 score (mean ± SD)	13.4 ± 4.1	13.3 ± 4.0	0.84
Participants with severe substance use disorder	136 (76.8)	199 (79.3)	0.627
History of treatment for substance use disorder	155 (87.6)	193 (76.9)	0.008
History of illicit drug use	166 (93.8)	240 (95.6)	0.533
History of prescription medication misuse	106 (59.9)	135 (53.8)	0.248
History of OTC drug misuse	64 (36.2)	86 (34.3)	0.763
Presence of a chronic disease	41 (23.2)	50 (19.9)	0.492
History of STBBIs	70 (39.5)	95 (37.8)	0.799
Heterosexual‐identifying participants	151 (85.3)	211 (84.1)	0.829

*Note*: Data are presented as *n* (%), unless otherwise indicated. Illicit drugs include cannabis, methamphetamine, cocaine, heroin, 3,4‐methylenedioxymethamphetamine (MDMA), and other novel psychoactive substances. “History of treatment for substance use disorder” refers to whether participants received any form of support or treatment for substance use disorder before entering the facility. “History of illicit drug use,” “history of prescription medication misuse,” and “history of OTC drug misuse” refer to whether participants had used or abused these substances before entering the facility. Statistical significance is set at *p* < 0.05.

Abbreviations: DAST‐20, Drug Abuse Screening Test‐20; OTC, over‐the‐counter; SD, standard deviation; STBBIs, sexually transmitted and blood‐borne infections.

However, the mean age at entry was comparable between the dual diagnoses and control cohorts (38.5 ± 9.7‐ and 38.9 ± 10.4‐year‐old, respectively), with no significant differences in education; employment, welfare, or legal statuses; or duration of facility utilization at baseline (32.7 ± 37.7 and 28.5 ± 35.6 months, respectively). Other facility‐related factors and health indicators, including drug dependence severity, presence of chronic illness, history of STBBIs, and sexual orientation, did not notably differ between the cohorts.

To ensure that cohort comparative analyses were not biased by differences in time since admission, we examined the proportion of participants who had been in the facility for <6 months and those for >3 years at baseline. These data are not included in Table 1. A total of 113 out of 428 participants had been in the facility for <6 months, with 43 (24.3%) and 70 (27.9%) in the dual diagnoses and control cohorts, respectively (*p* = 0.472). Additionally, 123 participants had been in the facility for >3 years, with 57 (32.2%) and 66 (26.3%) in the dual diagnoses and control cohorts, respectively (*p* = 0.222). Thus, no statistically significant differences were observed between the cohorts.

### Multivariable logistic regression analysis of the factors correlated with dual diagnoses

Table 2 presents the results of the multivariable logistic regression analysis, which included variables that were either statistically significant in Table 1 or clinically relevant, such as sex, age at entry into the facility, incarceration history, and prior treatment for substance use disorder. Male sex was correlated with lower odds of dual diagnoses (AOR = 0.24, 95% CI: 0.11–0.50), indicating a higher likelihood among the female participants. A history of incarceration (AOR = 2.10, 95% CI: 1.35–3.28) and prior treatment for substance use disorder (AOR = 2.22, 95% CI: 1.30–3.91) were both positively correlated with dual diagnoses. All associations remained significant after applying FDR correction (*q* < 0.05) (Table S1).

**Table 2:** Multivariable logistic regression analysis of factors associated with dual‑diagnosis status.

	AOR (95% CI)	*p* value
Female participant	4.18 (2.01–8.67)	<0.001
Age at entry to the facility, years	0.99 (0.97–1.01)	0.456
Criminal incarceration history	2.10 (1.35–3.28)	0.001
History of treatment for substance use disorder	2.22 (1.30–3.91)	0.004

*Note*: “History of treatment for substance use disorder” refers to whether participants received any form of support or treatment for substance use disorder before entering the facility. Statistical significance is set at *p* < 0.05.

Abbreviations: AOR, adjusted odds ratio; CI, confidence interval.

### Exploratory analysis of dual diagnoses and 1‐year recovery outcomes

Logistic regression analysis showed that participants with dual diagnoses experienced more challenges in recovery 1 year after entering DARC facilities (Table 3). Specifically, they were significantly more likely to be unemployed compared to those without dual diagnoses (AOR = 2.10, 95% CI: 1.35–3.31), and they had more difficulty maintaining abstinence from substance use (AOR = 1.85, 95% CI: 1.17–2.94). In contrast, there was no significant difference between the two groups in terms of welfare receipt (AOR = 0.98, 95% CI: 0.61–1.58). Of these outcomes, only the association with unemployment remained statistically significant after FDR correction (Table S2).

**Table 3:** Logistic regression analysis of the association between dual diagnoses and 1‐year recovery outcomes.

Recovery outcome	Variable	AOR (95% CI)	*p* value
Employment (no)	Dual diagnosis	2.10 (1.35–3.31)	0.001
	Female participant	0.94 (0.45–2.04)	0.869
	Age at entry into the facility	1.03 (1.00–1.05)	0.026
	Criminal incarceration history	1.49 (0.95–2.33)	0.083
	History of treatment for substance use disorder	1.47 (0.87–2.47)	0.145
Receiving welfare (yes)	Dual diagnosis	0.98 (0.61–1.58)	0.939
	Female participant	0.88 (0.41–1.96)	0.744
	Age at entry into facility	1.02 (0.99–1.04)	0.137
	Criminal incarceration history	1.67 (1.02–2.74)	0.042
	History of treatment for substance use disorder	1.64 (0.94–2.84)	0.078
Sustained abstinence (no)	Dual diagnosis	1.85 (1.17–2.94)	0.008
	Female participant	1.12 (0.51–2.38)	0.770
	Age at entry into facility	0.99 (0.97–1.01)	0.424
	Criminal incarceration history	1.01 (0.62–1.64)	0.964
	History of treatment for substance use disorder	1.15 (0.64–2.15)	0.643

*Note*: “History of treatment for substance use disorder” refers to whether participants received any form of support or treatment for substance use disorder before entering the facility. Statistical significance is set at *p* < 0.05.

Abbreviations: AOR, adjusted odds ratio; CI, confidence interval.

### Sub‐cohort analysis by sex: Factors correlated with dual diagnoses

The results of the sub‐cohort analysis by sex are presented in Tables S3–S6. For male participants, the multivariable logistic regression revealed that a history of incarceration (AOR = 2.24, 95% CI: 1.41–3.60) and prior support or treatment for a substance use disorder (AOR = 2.43, 95% CI: 1.37–4.47) were statistically significantly correlated with a higher odds of dual diagnoses (Table S5). Among the female participants, older age at facility entry was considerably correlated with dual diagnoses (AOR = 1.17, 95% CI: 1.04–1.40) (Table S6).

## DISCUSSION

### Correlation between dual diagnoses and sex

A key strength of this study is its focus on DARC residents, who are already engaged in a structured, peer‐led recovery system. We found that females in drug recovery facilities were more likely than males to have dual diagnoses. This contrasts with population‐based studies, which typically report higher rates among males, especially those with externalizing disorders such as antisocial personality disorder or schizophrenia.^2^

This difference may reflect variations in care pathways between sexes in DARC. Men often enter support systems via the justice route,^3^ whereas women more commonly connect through medical or welfare services owing to trauma or family circumstances.^40^, ^41^, ^42^ Previous work corroborates this pattern, showing that women with dual diagnoses frequently report physical or sexual abuse, posttraumatic stress disorder, depression, or suicide attempts.^40^, ^41^, ^42^ Consequently, many females may enter recovery facilities already having been identified with dual diagnoses. Moreover, due to the limited number of female‐only recovery facilities in Japan, entry into rehabilitation facilities may have been prioritized for those with particularly severe conditions. This potentially resulted in a higher proportion of female participants with serious mental health and substance use problems in the study population, contributing to the observed findings.

The sex‐stratified analyses further supported these findings. Among male participants, dual diagnoses were correlated with incarceration and prior treatment, patterns related to impulsive or externalizing psychiatric traits that prompted earlier contact with the justice system^2^ and increased the chances of diagnosis.^25^ Among the female participants, only older age at facility entry was notably associated, suggesting that their dual diagnoses may develop or be recognized after years of delayed help‐seeking due to caregiving or family roles.^3^, ^20^, ^43^ Trauma; life events, such as menopause or bereavement; and gradual exposure to support services may have contributed to the complexity of their conditions over time. These findings highlight the importance of incorporating sex‐sensitive frameworks into recovery support systems. Accounting for the distinct risk profiles and treatment trajectories of males and females may enhance the effectiveness of interventions and promote more equitable recovery outcomes within Japan's rehabilitation landscape.

### Correlation between dual diagnosis and incarceration history

In this study, among the participants using drug rehabilitation support facilities, a history of incarceration showed a notable correlation with a dual diagnosis. Conversely, no clear association was observed between a dual diagnosis and legal status at the start of facility utilization. This difference may reflect the distinct implications of the two variables. Legal status represents a temporary condition, whereas incarceration history may reflect cumulative effects more closely related to dual diagnoses, such as repeated behavioral patterns, limited access to support, and sustained involvement within the justice system.

Individuals with dual diagnoses are prone to impulsiveness and impaired judgment because of the interaction between psychiatric symptoms and substance use, which may elevate the risk of reoffending or reincarceration.^44^, ^45^ Consequently, dual diagnoses may be more closely associated with enduring behavioral patterns captured by incarceration history than with the temporary conditions reflected by legal status.

Under Japan's current legal framework, mechanisms such as suspended sentences and probation provide individuals with substance use or mental disorders with opportunities to access community‐based support.^46^, ^47^ Correctional facilities, including prisons and detention centers, may play a key role in assessing mental health and initiating treatment, serving as an entry point to care. In particular, regarding individuals with drug‐related offenses and psychiatric conditions, contact with the justice system generally precedes any medical or welfare involvement, with diagnosis and support typically beginning after incarceration.

By contrast, individuals without a history of incarceration who enter rehabilitation facilities through less severe legal dispositions, such as probation or deferred prosecution, may not undergo adequate psychiatric evaluation. Thus, they may not be diagnosed with dual diagnoses. This could partly explain the lack of correlation between the legal status and a dual diagnosis in our study. These findings suggest a need to establish mechanisms that facilitate earlier access to support for individuals with dual diagnoses.

### Correlation between dual diagnoses and history of treatment for substance use disorders

Residents with a dual diagnosis had significantly higher odds of having accessed medical or welfare services for substance use disorders before entering a DARC. This association must be interpreted in light of how dual diagnosis was ascertained in our study. Because comorbid psychiatric disorders were identified retrospectively from existing medical records rather than through contemporaneous structured interviews, individuals who interacted more often with healthcare providers simply had more opportunities to receive additional diagnoses. In other words, the pattern we observed may partly reflect a “diagnostic‑opportunity” bias, not necessarily greater intrinsic clinical severity.

### Correlation between dual diagnoses and 1‐year follow‐up outcomes

Compared with participants without dual diagnoses, those with dual diagnoses were less likely to be employed and had greater difficulty maintaining abstinence from substance use. These findings align with trends reported in previous studies.^48^, ^49^ Individuals with dual diagnoses frequently encounter greater barriers to stable employment, sustained abstinence, and ongoing participation in recovery programs. Research suggests that the complex interplay between psychiatric symptoms and substance use can disrupt motivation, emotional regulation, and adherence to treatment regimens. Furthermore, instability in social relationships and difficulty managing daily stressors may further undermine recovery efforts.

However, after applying FDR correction for multiple comparisons, only the association between dual diagnosis and employment remained statistically significant. Associations with abstinence and other outcomes were no longer significant. This suggests that although dual diagnoses may broadly impact functioning, the most reliable evidence in this study points to employment‐related difficulties.

These findings suggest that recovery facilities should provide integrated programs that address both mental health and substance use disorders concurrently. Adapting flexible plans to match the needs of people with dual diagnoses might improve effectiveness and help staff enhance the practical delivery of recovery support.

### Study limitations

First, dual diagnoses were self‐reported rather than clinically verified, making misclassification likely. If misclassification were nondifferential—assuming Se = 0.65 and Sp = 0.90 for both groups^50^, ^51^—the corrected odds ratio would be about 3.6, higher than the observed 2.2.^52^ By contrast, in our study, diagnostic accuracy may vary by treatment history, as previously treated individuals are more likely to have received and be aware of a formal diagnosis. This differential misclassification could bias results in either direction. For example, holding specificity at 0.90 and allowing the sensitivity to range from 0.35 to 0.65 yields corrected odds ratios from 0.7 to 3.6, indicating the association could weaken, disappear, or even reverse. Given these possibilities, the magnitude and direction of the association remain uncertain, and results should be interpreted with caution. Second, we lacked data on disorder severity, onset, and specific diagnostic categories, preventing subtype analyses. Third, although the overall sample size was adequate, the number of female participants was small, potentially limiting the statistical power of sex‐stratified analyses, particularly among participants of this sub‐cohort. Finally, the 1‐year follow‐up period captured only short‐term outcomes, without assessing long‐term recovery or relapse over several years. However, to the best of our knowledge, this is the longest follow‐up study of individuals in drug recovery facilities conducted in Japan, offering rare and valuable insights into this high‐risk population.

### Concluding statement

This study identified the key characteristics correlated with dual diagnoses among drug rehabilitation support facility members in Japan. A dual diagnosis was correlated with the female sex, a history of incarceration, and prior treatment experience, with different factors associated with dual diagnoses for each sex. Participants with dual diagnoses experienced poorer recovery outcomes, including lower employment rates and greater difficulty maintaining abstinence from substance use, compared with those without a dual diagnosis. These correlations should be viewed as opportunities to address substance use disorders and conduct comprehensive assessments that encompass mental health. Such an approach could facilitate earlier identification and intervention for individuals with dual diagnoses.

## AUTHOR CONTRIBUTIONS

Takuya Shimane designed the preliminary experiments and established the participant database. Takuya Shimane and Satoshi Inoura recruited participants and collected data. Toshihiko Matsumoto and Takuya Shimane secured funding. Satomi Mizuno designed the study, performed statistical analyses, and drafted the initial manuscript. Takuya Shimane, Satoshi Inoura, and Toshihiko Matsumoto supervised the manuscript preparation. All authors revised, reviewed, and approved the final version of the manuscript for publication.

## CONFLICT OF INTEREST STATEMENT

The authors declare no conflicts of interest.

## ETHICS APPROVAL STATEMENT

The original study^29^, ^35^ was approved by the Ethics Committee of the National Centre of Neurology and Psychiatry, Japan (approval number: A2016‐022). This study was conducted in accordance with the principles of the Declaration of Helsinki and STROBE reporting guidelines.

## PATIENT CONSENT STATEMENT

Patients were informed regarding the purpose of the study, and those who expressed interest were invited to participate after providing written informed consent. Written informed consent was obtained from all study participants for both the original and present studies.

## CLINICAL TRIAL REGISTRATION

This observational study did not qualify as a clinical trial under the guidelines established by the International Committee of Medical Journal Editors.

## Supporting information


Supporting Information.

## Data Availability

Due to ethical and legal restrictions, the data used in this study are not publicly available.
