# Supplementary material for: Co‑occurring mental and substance use disorders among residents of Drug Addiction Rehabilitation Centers (DARCs) in Japan: Characterizing dual‑diagnosis profiles
Source: PCN Rep. 2025 Sep 24;4(3):e70196. doi: 10.1002/pcn5.70196 (PMC12458395; doi:10.1002/pcn5.70196)
Supplement: Supplementary file 1 — Supporting Information. [file PCN5-4-e70196-s001.docx]

**Supporting information**

**Supplementary Table 1. FDR–adjusted q-values for multivariable logistic regression results presented in Table 2**

|  | AOR (95% CI) | p-value | q-value |
| --- | --- | --- | --- |
| Female participant | 4.18 (2.01–8.67) | <0.001 | 0.001 |
| Age at entry to the facility, years | 0.99 (0.97ー1.01) | 0.456 | 0.456 |
| Criminal incarceration history | 2.10 (1.35ー3.28) | 0.001 | 0.002 |
| History of treatment for substance use disorder | 2.22 (1.30ー3.91) | 0.004 | 0.005 |

Abbreviations: AOR, adjusted odds ratio; CI, confidence interval.

“History of treatment for substance use disorder” refers to whether participants received any form of support or treatment for substance use disorder prior to entering the facility.

The Benjamini-Hochberg method was applied to control for the false discovery rate (FDR). Variables with q < 0.05 were considered statistically significant after correction.

**Supplementary Table 2. FDR–adjusted q-values for multivariable logistic regression results presented in Table 3**

| Recovery outcome | Variable | AOR (95% CI) | p-value | q-value |
| --- | --- | --- | --- | --- |
| Employment (No) | Dual diagnosis | 2.10 (1.35–3.31) | 0.001 | 0.015 |
|  | Male participant | 1.06 (0.49–2.23) | 0.869 | 0.964 |
|  | Age at entry into the facility | 1.03 (1.00–1.05) | 0.026 | 0.129 |
|  | Criminal incarceration history | 1.49 (0.95–2.33) | 0.083 | 0.207 |
|  | History of treatment for substance use disorder | 1.47 (0.87–2.47) | 0.145 | 0.271 |
| Receiving welfare (Yes) | Dual diagnosis | 0.98 (0.61–1.58) | 0.939 | 0.964 |
|  | Male participant | 1.13 (0.51–2.41) | 0.744 | 0.962 |
|  | Age at entry into facility | 1.02 (0.99–1.04) | 0.137 | 0.271 |
|  | Criminal incarceration history | 1.67 (1.02–2.74) | 0.042 | 0.157 |
|  | History of treatment for substance use disorder | 1.64 (0.94–2.84) | 0.078 | 0.207 |
| Sustained abstinence (No) | Dual diagnosis | 1.85 (1.17–2.94) | 0.008 | 0.060 |
|  | Male participant | 0.89 (0.42–1.97) | 0.770 | 0.962 |
|  | Age at entry into facility | 0.99 (0.97–1.01) | 0.424 | 0.706 |
|  | Criminal incarceration history | 1.01 (0.62–1.64) | 0.964 | 0.964 |
|  | History of treatment for substance use disorder | 1.15 (0.64–2.15) | 0.643 | 0.962 |

Abbreviations: AOR, adjusted odds ratio; CI, confidence interval.

“History of treatment for substance use disorder” refers to whether participants received any form of support or treatment for substance use disorder prior to entering the facility.

The Benjamini-Hochberg method was applied for the false discovery rate (FDR). Variables with q < 0.05 were considered statistically significant after correction.

**Supplementary Table 3. Descriptive statistics of male participants by dual diagnosis status**

|  | Dual diagnosis cohort (n = 151) | Non-dual diagnosis (control)  cohort (n = 239) | p-value |
| --- | --- | --- | --- |
| Age at entry into the facility (mean ± SD), years | 39.26 ± 9.78 | 39.41 ± 10.13 | 0.783 |
| Did not complete high school | 59 (39.1) | 115 (48.1) | 0.1 |
| Employment | 22 (14.6) | 46 (19.2) | 0.294 |
| Welfare recipient | 131 (86.8) | 195 (81.6) | 0.23 |
| Criminal incarceration history | 97 (64.2) | 116 (48.5) | 0.003 |
| Legal status at facility entry | 64 (42.4) | 99 (41.4) | 0.935 |
| Participants utilizing residential rehabilitation services | 131 (86.8) | 218 (91.2) | 0.219 |
| Duration of facility utilization prior to baseline survey (mean ± SD), months | 33.1 ± 37.6 | 28.3 ± 35.5 | 0.205 |
| Active participation in rehabilitation programs | 130 (86.1) | 196 (82.0) | 0.357 |
| Positive relationships with other residents and facility staff | 144 (95.4) | 231 (96.7) | 0.708 |
| Presence of a sponsor | 116 (76.8) | 186 (77.8) | 0.915 |
| Severity at facility entry: DAST-20 score (mean ± SD) | 13.45 ± 4.09 | 13.36 ± 3.96 | 0.828 |
| Participants with severe substance use disorder | 115 (76.2) | 189 (79.1) | 0.581 |
| History of treatment for substance use disorder | 133 (88.1) | 182 (76.2) | 0.005 |
| History of illicit drug use | 143 (94.7) | 228 (95.4) | 0.945 |
| History of prescription medication misuse | 86 (57.0) | 125 (52.3) | 0.427 |
| History of OTC drug misuse | 51 (33.8) | 80 (33.5) | 1 |
| Presence of a chronic disease | 35 (23.2) | 50 (20.9) | 0.689 |
| History of STBBIs | 59 (39.1) | 92 (38.5) | 0.994 |
| Heterosexual-identifying participants | 128 (84.8) | 203 (84.9) | 1 |

Data are presented as n (%), unless otherwise indicated.

Illicit drugs include cannabis, methamphetamine, cocaine, heroin, 3, 4-methylenedioxymethamphetamine (MDMA), and other novel psychoactive substances.

Abbreviations: SD, standard deviation; DAST-20, Drug Abuse Screening Test 20; STBBIs, sexually transmitted and blood-borne infections; OTC, over-the-counter.

“History of treatment for substance use disorder” refers to whether participants received any form of support or treatment for substance use disorder prior to entering the facility.

“History of illicit drug use,” “history of prescription medication misuse,” and “history of OTC drug misuse” refer to whether participants had used or abused these substances prior to entering the facility.

Statistical significance was set at p < 0.05.

**Supplementary Table 4**. **Descriptive statistics of female participants by dual diagnosis status**

|  | Dual diagnosis cohort (n = 26) | Non-dual diagnosis (control)  cohort (n = 12) | p-value |
| --- | --- | --- | --- |
| Age at entry into the facility (mean ± SD), years | 34.04 ± 7.72 | 27.83 ± 8.50 | 0.032 |
| Did not complete high school | 16 (61.5) | 6 (50.0) | 0.752 |
| Employment | 6 (23.1) | 1 (8.3) | 0.522 |
| Welfare recipient | 22 (84.6) | 8 (66.7) | 0.405 |
| Criminal incarceration history | 6 (23.1) | 2 (16.7) | 0.982 |
| Legal status at facility entry | 8 (30.8) | 4 (33.3) | 1 |
| Participants utilizing residential rehabilitation services | 17 (65.4) | 9 (75.0) | 0.828 |
| Duration of facility utilization prior to baseline survey (mean ± SD), months | 30.38 ± 38.64 | 31.25 ± 39.71 | 0.950 |
| Active participation in rehabilitation programs | 25 (96.2) | 9 (75.0) | 0.160 |
| Positive relationships with other residents and facility staff | 24 (92.3) | 11 (91.7) | 1 |
| Presence of a sponsor | 24 (92.3) | 9 (75.0) | 0.342 |
| Severity at facility entry: DAST-20 score (mean ± SD) | 13.15 ± 4.46 | 12.67 ± 4.54 | 0.757 |
| Participants with severe substance use disorder | 21 (80.8) | 10 (83.3) | 1 |
| History of treatment for substance use disorder | 22 (84.6) | 11 (91.7) | 0.935 |
| History of illicit drug use | 23 (88.5) | 12 (100.0) | 0.563 |
| History of prescription medication misuse | 20 (76.9) | 10 (83.3) | 0.982 |
| History of OTC misuse | 13 (50.0) | 6 (50.0) | 1 |
| Presence of chronic disease | 6 (23.1) | 0 (0.0) | 0.182 |
| History of STBBIs | 11 (42.3) | 3 (25.0) | 0.505 |
| Heterosexual-identifying participants | 23 (88.5) | 8 (66.7) | 0.246 |

Data are presented as n (%), unless otherwise indicated.

Illicit drugs include cannabis, methamphetamine, cocaine, heroin, 3, 4-methylenedioxymethamphetamine (MDMA), and other novel psychoactive substances.

Abbreviations: SD, standard deviation; DAST-20, Drug Abuse Screening Test 20; STBBIs, sexually transmitted and blood-borne infections; OTC, over-the-counter.

“History of treatment for substance use disorder” refers to whether participants received any form of support or treatment for substance use disorder prior to entering the facility.

“History of illicit drug use,” “history of prescription medication misuse,” and “history of OTC drug misuse” refer to whether participants had used or abused these substances prior to entering the facility.

Statistical significance was set at p < 0.05.

**Supplementary Table 5. Multivariable logistic regression analysis of factors correlated with dual diagnosis status among male participants**

|  | AOR (95% CI) | p-value |
| --- | --- | --- |
| Age at entry into the facility, years | 0.99 (0.96–1.01) | 0.217 |
| Criminal incarceration history | 2.24 (1.41–3.60) | <0.001 |
| History of treatment for substance use disorder | 2.43 (1.37–4.47) | 0.003 |

Abbreviations: AOR, adjusted odds ratio; CI, confidence interval.

The reference for the participant’s facility utilization status is based on their current residence.

“History of treatment for substance use disorder” refers to whether participants had used or abused these substances prior to entering the facility.

Statistical significance was set at p < 0.05.

**Supplementary Table 6. Multivariable logistic regression analysis of factors correlated with dual diagnosis status among female participants**

|  | AOR (95% CI) | p-value |
| --- | --- | --- |
| Age at entry into facility | 1.17 (1.04–1.40) | 0.033 |
| Criminal incarceration history | 1.64 (0.18–24.57) | 0.676 |
| History of treatment for substance use disorder | 0.21 (0.01–2.25) | 0.255 |

Abbreviations: AOR, adjusted odds ratio; CI, confidence interval.

The reference for the participant’s facility utilization status is based on their current residence.

“History of treatment for substance use disorder” refers to whether participants had used or abused these substances prior to entering the facility.

Statistical significance was set at p < 0.05.
